# Low Dose Rivaroxaban for Atherosclerotic Cardiovascular Diseases: A Systematic Review and Meta-analysis

**DOI:** 10.3389/fphar.2020.608247

**Published:** 2021-02-08

**Authors:** Can Chen, Yuanqing Kan, Zhenyu Shi, Daqiao Guo, Weiguo Fu, Yanli Li, Qianzhou Lv, Xiaoyu Li, Yi Si

**Affiliations:** ^1^ Department of Pharmacy, Zhongshan Hospital, Fudan University, Shanghai, China; ^2^ Department of Vascular Surgery, Zhongshan Hospital, Fudan University, Shanghai, China

**Keywords:** rivaroxaban, low dose, atherosclerosis, cardiovascular diseases, systematic review, randomized controlled studies

## Abstract

**Background:** This study aims to explore the role of low-dose rivaroxaban (≤10 mg daily) for the treatment of atherosclerotic cardiovascular disease (ASCVD).

**Methods:** PubMed, Embase and the Cochrane Library were searched for randomized controlled trials (RCTs) of low-dose rivaroxaban in patients with ASCVD including coronary artery disease (CAD) and peripheral artery disease (PAD). Literature screening, data extraction, and risk of bias assessment were carried out independently by two researchers. Hazard ratio (HR) and 95% confidence interval (CI) were calculated using random-effect models to determine risks of outcomes in ASCVD patients treated with rivaroxaban and comparators, and meta-analysis was conducted via Review Manager 5.3.5 software.

**Results:** 3,768 records were obtained through literature search, and 9 articles representing 6 RCTs ultimately qualified for this study. The meta-analysis indicated that for patients with CAD, the addition of rivaroxaban (5 mg daily) to aspirin could significantly reduce the risk of major adverse cardiovascular events (MACEs) compared with aspirin alone (HR 0.81, 95% CI, 0.72 to 0.91, *p* = 0.0004, I^2^ = 60%, 4 studies). For PAD patients receiving rivaroxaban (5 mg daily) plus aspirin, there was no significant reduction in the risk of MACEs (HR 0.84, 95% CI, 0.63 to 1.13, *p* = 0.25, I^2^ = 74%, 2 studies); however, there was significant reduction in major adverse limb events (MALEs) (HR 0.54, 95% CI, 0.35 to 0.83, *p* = 0.005, one studies) and in the composite of MACEs or MALEs (HR 0.78, 95% CI, 0.64 to 0.95, *p* = 0.02, I^2^ = 66%, 2 studies) when compared with patients receiving aspirin alone. Meanwhile, rivaroxaban combined with aspirin significantly increased the risk of International Society on Thrombosis and Haemostasis (ISTH) major bleeding compared with aspirin alone in patients with CAD (HR 1.74, 95% CI, 1.43 to 2.13, *p* < 0.00001, I^2^ = 0%, 2 studies) and PAD (HR 1.47, 95% CI, 1.19 to 1.83, *p* = 0.0004, I^2^ = 0%, 2 studies).

**Conclusions:** Compared with standard antiplatelet therapy, the addition of a 5 mg daily dose of rivaroxaban to standard antiplatelet therapy may improve cardiovascular or limb outcomes of patients with ASCVD, with an increase in major bleeding. Patients who would benefit from the addition of low-dose rivaroxaban to antiplatelet agents and appropriate dual-pathway antithrombotic strategies should be identified in clinical practice to individualize antithrombotic therapy.

## Introduction

Aspirin is considered the cornerstone of secondary prevention based on numerous studies consistently demonstrating that aspirin alone or in combination with P2Y12 inhibition can significantly reduce ischemic events and cardiovascular (CV) deaths resulting from acute coronary syndrome (ACS) (Mehta et al., 2010), stable coronary artery disease (CAD) (Antithrombotic Trialists’ Collaboration, 2002; Udell et al., 2016), and peripheral artery disease (PAD) (Beiswenger et al., 2018). However, 5–10% of patients with atherosclerotic cardiovascular disease (ASCVD) patients experience recurring CV events every year (Bhatt et al., 2010). Although a variety of strategies have been explored to improve long-term outcomes of acetylsalicylic acid (ASA) therapy (CAPRIE Steering Committee, 1996; Bhatt et al., 2006; Bonaca et al., 2015; Hiatt et al., 2017; Bhatt et al., 2019; Mehran et al., 2019), optimal long-term antithrombotic therapy for chronic vascular diseases have yet to be determined (Gurbel et al., 2019).

Recently, more attention has been focused on the efficacy and safety of dual-pathway inhibition strategies which combine antiplatelet agents and Direct oral anticoagulants (DOACs) for preventing CV events in ASCVD. DOACs inhibit the thrombin pathway activity that amplifies platelet activation through inhibition of thrombin generation by targeting factor Xa (Gurbel and Tantry, 2010). Rivaroxaban is the first oral direct activated factor Xa inhibitor to be approved for use (Samama, 2011) and has been the most widely studied option for ASCVD therapy (Mega et al., 2009; Mega et al., 2012; Eikelboom et al., 2017; Ohman et al., 2017; Zannad et al., 2018). The renowned Cardiovascular Outcomes for People Using Anticoagulation Strategies (COMPASS) trial (Eikelboom et al., 2017) revealed that for stable CAD and PAD participants, rivaroxaban (2.5 mg twice a day) plus ASA could reduce the risk of major adverse cardiovascular events (MACEs) in comparison with ASA alone, and occurrences of MACEs were not significantly different between rivaroxaban (5 mg twice a day) and ASA alone. A recent systemic review (Khan et al., 2020) which included 5 randomized controlled trials (RCTs) found that for CAD patients, low-dosage rivaroxaban (2.5 mg twice daily) was associated with a reduction in myocardial infarction (MI) and stroke at the expense of major bleeding when compared with the controls. Based on the COMPASS trial, the newly published Vascular Outcomes Study of ASA (acetylsalicylic acid) along with Rivaroxaban in Endovascular or Surgical Limb Revascularization for PAD (peripheral artery disease) (VOYAGER PAD) (Bonaca et al., 2020) has indicated that compared with aspirin alone, rivaroxaban (2.5 mg twice a day) combined with aspirin decreased the risk of composite outcome of MACEs or MALEs in PAD patients after lower-extremity revascularization.

Since previous systematic reviews (Obonska et al., 2013; Oldgren et al., 2013; Villablanca et al., 2017; Yuan, 2018; Khan et al., 2020) did not include PAD population data in the analysis, and only focused on comparisons between rivaroxaban plus aspirin versus aspirin alone, our study aims to investigate and analyzed the efficacy and safety of low-dosage rivaroxaban (5 mg or 10 mg daily dose) with or without aspirin in ASCVD, including CAD and PAD patients.

## Methods

This work was performed according to the Preferred Reporting Items for Systematic Review and Meta-Analyses (PRISMA) guidelines (Liberati et al., 2009). The PROSPERO registration number of this study protocol is CRD42020179891.

### Search Strategy

We systematically searched PubMed, Embase and Cochrane Library databases for RCTs published through April 26, 2020. Furthermore, a complementary search was conducted in a similar fashion and additional papers published through August 31, 2020 were added to the literature list. In this review, PAD is considered as lower extremity peripheral artery disease or carotid artery disease. The following medical subject headings were applied to make a combination: “Rivaroxaban,” “Acute Coronary Syndrome,” “Peripheral arterial disease,” “Arterial Occlusive Diseases,” “Carotid Artery Diseases,” “Coronary Artery Disease,” “Carotid Stenosis,” and free terms including “Rivaroxaban,” “Xarelto,” “Acute Coronary Syndrome,” “Peripheral arterial disease,” “Arterial Occlusive Diseases,” “Carotid Artery Diseases,” “Coronary Artery Disease,” “Carotid Stenosis,” “Random,” “Randomization,” “Randomised,” “Randomized,” and “Randomly.” Supplementary Tables S1–S3 show search strategies of the above three databases. Moreover, we checked references listed in included articles for additional qualifiers.

### Study Selection

Studies were included if they were published in English and enrolled ASCVD patients (age ≥18 years) prescribed with low-dose rivaroxaban (≤10 mg daily), and. Articles on post-hoc and subgroup analysis of included studies were also eligible if they reported predefined outcomes. We excluded works that did not report outcomes of interest, were not published as full texts, and only focused on patients all administrated with rivaroxaban at dosages higher than 10 mg per day.

MACE, which is defined as the composite of MI, stroke, or CV death, is a commonly used end point for CV research (Kip et al., 2008; Hirshberg and Katz, 2013) and was selected as the primary efficacy outcome for the entire ASCVD population. Key composite outcomes for PAD were MALE, defined as the composite of acute limb ischemia (ALI), chronic limb ischemia (CLI) or major amputation, and the composite of MACE or MALE. The secondary efficacy outcomes were MI, stroke, and CV death for all ASCVD patients, and ALI, CLI, and major amputation for PAD. As with most antithrombotic agent trials (Mega et al., 2012; Eikelboom et al., 2017; Zannad et al., 2018; Bonaca et al., 2020), the safety endpoint of interest was major bleeding. International Society on Thrombosis and Haemostasis (ISTH) major bleeding (Schulman and Kearon, 2005) and Thrombolysis in Myocardial Infarction (TIMI) classification major bleeding (Rao et al., 1988; Bovill et al., 1991) were assessed as primary and secondary safety outcomes, respectively. We adopted definitions used in original studies for the remaining outcomes.

Two reviewers (C.C and YQ. K) conducted the study selection independently, and a third reviewer (Y.S or XY. L) was involved in the event of any discrepancies.

### Data Abstraction

Two reviewers (C.C and YQ.K) independently abstracted recorded data from the included studies using a standardized Excel table. The table includes four parts: 1) study details (such as authors, year of publication, country, region, and blinding); 2) characteristics of the included population (such as diagnosis, age, and sex); 3) treatment protocols including antiplatelet regimens, rivaroxaban dosage and follow-up time; and 4) efficacy and safety outcomes. Y.S or XY. L would provide resolution assistance when C.C and YQ. K disagreed on differences in data extraction. If a study had two or more intervention groups, we combined them into a single new intervention group for primary analysis, and selected the corresponding intervention group for subgroup analysis (Higgins et al., 2019). For example, patients enrolled in the ATLAS ACS 2-TIMI 51 (Mega et al., 2012) and treated with standard therapy were randomly assigned to receive rivaroxaban 2.5 mg twice daily (Group A), rivaroxaban 5 mg twice daily (Group B), or placebo twice daily; Groups A and B would be merged into a new group (Group C) for primary analysis and Group A or Group B would been selected for subgroup analysis based on the rivaroxaban dosage.

### Risk of Bias Assessment and Quality of the Evidence

We evaluated risks of bias using the Cochrane Collaboration’s tool, which assesses risk of bias in randomized trials (Higgins et al., 2019) and is comprised of the following 7 domains: random sequence generation, allocation concealment, blinding of participants and personnel, blinding of outcome assessment, incomplete outcome data, selective reporting, and anything else.

The Grading of Recommendations Assessment, Development and Evaluation (GRADE) approach was adopted to assess quality of evidence. GRADE assessments include 5 domains for rating down (risk of bias, inconsistency, indirectness, imprecision and publication bias) and 3 domains for rating up (large magnitude of effect, dose-response gradient, and decrease in magnitude of effect due to residual confounding) (Higgins et al., 2019). The GRADE approach divides quality of evidence into high, moderate, low, and very low level (Higgins et al., 2019).

### Statistical Analysis

We calculated the hazard ratio (HR) and 95% confidence interval (CI) using random-effect models for incidence of outcomes in ASCVD patients, and Review Manager 5.3.5 software (Review Manager, 2014) was employed for the meta-analysis. χ2 test and I2 statistics were used to explore heterogeneity, and I^2^ values of 25, 50, and 75% represented low, moderate, and high heterogeneity, respectively (Higgins et al., 2003). For primary outcomes reported by three or more studies, subgroup analyses were performed by grouping eligible patients according to the their diagnosis (ACS, stable CAD, or PAD), daily rivaroxaban dosage (5 mg or 10 mg per day), and antiplatelet regimen (single or dual antiplatelet therapy). We conducted sensitivity analysis by altering the effect models and individually eliminating studies during data synthesis of the three or more studies reporting primary outcomes. A p-value of less than 0.05 was considered as a statistically significant difference. Moreover, GRADEpro Guideline Development Tool (GDT) (https://gradepro.org/) was used to determine quality of evidence and calculate the number needed to treat (NNT) for primary and key composite outcomes.

## Results

### Literature Search

**FIGURE 1:**
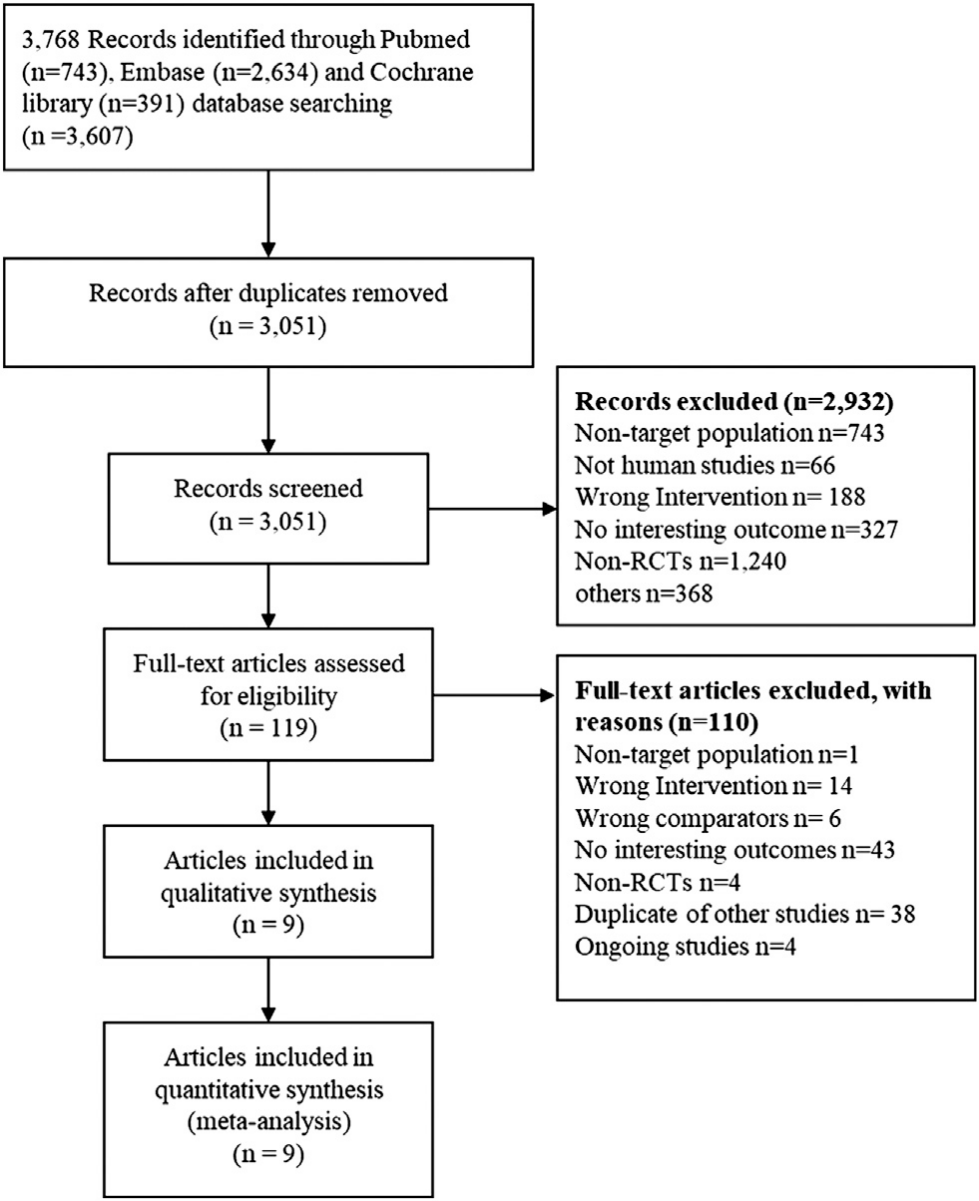
The PRISMA 2009 flow diagram for literature screening. RCTs randomized controlled trials.

Out of the 3,768 identified articles, 119 potential articles were selected, and 9 English articles (Mega et al., 2009; Mega et al., 2012; Eikelboom et al., 2017; Ohman et al., 2017; Anand et al., 2018; Connolly et al., 2018; Zannad et al., 2018; Greenberg et al., 2019; Bonaca et al., 2020) published between 2012–2020 representing 6 RCTs (Mega et al., 2009; Mega et al., 2012; Eikelboom et al., 2017; Ohman et al., 2017; Zannad et al., 2018; Bonaca et al., 2020) were ultimately eligible. Three of the articles (Eikelboom et al., 2017; Anand et al., 2018; Connolly et al., 2018) were from the COMPASS trial, and two came from the COMMANDER HF trial (Zannad et al., 2018; Greenberg et al., 2019). The PRISMA 2009 flow diagram for the study selection is shown in Figure 1.

### Characteristics and Risk Bias of the Eligible Studies

Of the 60,067 eligible patients in the 6 RCTs (Mega et al., 2009; Mega et al., 2012; Eikelboom et al., 2017; Ohman et al., 2017; Zannad et al., 2018; Bonaca et al., 2020), 21,945 received rivaroxaban 5 mg per day, 15,349 received rivaroxaban 10 mg per day, and 22,773 received a placebo or aspirin. All studies were double-blind and conducted at multiple centers. Five studies compared rivaroxaban plus aspirin to aspirin alone, and two studies (Eikelboom et al., 2017; Ohman et al., 2017) compared rivaroxaban to aspirin with (Ohman et al., 2017) or without (Eikelboom et al., 2017) P2Y12 inhibitors. A portion of patients from four studies (Mega et al., 2009; Mega et al., 2012; Ohman et al., 2017; Bonaca et al., 2020) were treated with P2Y12 inhibitors including clopidogrel, ticagrelor, or ticlopidine. The subjects included 21,086 ACS patients (Mega et al., 2009; Mega et al., 2012; Ohman et al., 2017), 29,846 stable CAD patients (Connolly et al., 2018; Zannad et al., 2018), and 14,043 PAD patients (Anand et al., 2018; Bonaca et al., 2020), and the mean or median age was 57 through 68 years old. Males accounted for 74 to 78% of all patients from the eligible reports. The mean or median follow up time for each study ranged from 6 to 28 months. The characteristics of the eligible articles are presented in Table 1.

**TABLE 1:** The basic characteristics of the eligible studies.

Study	Year	Country (Center)	Study period	Population	The use of thienopyridine (percent)	Intervention	Sample	Age (years)^c^	Male	Follow-up time	Primary efficacy end point
VOYOGER PAD Bonaca et al. (2020)	2020	34 (542)	Aug 2015 to Jan 2018	PAD after revascularization	Clopidogrel (50%)^a^	Rivaroxaban 2.5 mg twice daily + aspirin 100 mg once daily	3,286	67.0 (61.0 to 73.0)	74%	Median 28 months	Composite of acute limb ischemia, major amputation for vascular causes, MI, ischemic stroke, or CV
						Placebo + aspirin 100 mg once daily	3,278	67.0 (61.0 to 73.0)	74%		
COMPASS Eikelboom et al. (2017)	2017	33 (602)	Mar 2013 to May 2016	Stable atherosclerotic vascular disease	Thienopyridine (0%)	Rivaroxaban 2.5 mg twice daily + aspirin 100 mg once daily	9,152	68.3 ± 7.9	77%	Mean 23 months	Composite of CV, stroke, or MI
						Rivaroxaban 5 mg twice daily + placebo	9,117	68.2 ± 7.9	78%		
						Aspirin 100 mg once daily + placebo	9,126	68.2 ± 7.9	78%		
COMPASS CAD Connolly et al. (2018)	2018	33 (602)	Mar 2013 to May 2016	Stable CAD	Thienopyridine (0%)	Rivaroxaban 2.5 mg twice daily + aspirin 100 mg once daily	8,313	69.0 (65.0 to 73.0)	79%	Mean 1.95 years	Composite of CV, stroke, or MI
						Rivaroxaban 5 mg twice daily + placebo	8,261	69.0 (65.0 to 73.0)	80%		
						Aspirin 100 mg once daily + placebo	8,250	69.0 (65.0 to 73.0)	80%		
COMPASS PAD Anand et al. (2018)	2018	33 (602)	Mar 2013 to May 2016	Stable peripheral or carotid artery disease	Thienopyridine (0%)	Rivaroxaban 2.5 mg twice daily + aspirin 100 mg once daily	2,474	67.9 ± 8.5	71%	Median 21 months	Composite of CV, stroke, or MI
						Rivaroxaban 5 mg twice daily + placebo	2,504	67.8 ± 8.5	73%		
						Aspirin 100 mg once daily + placebo	2,492	67.8 ± 8.5	71%		
COMMANDER HF Zannad et al. (201)	2018	32 (628)	Sep 2013 to Oct 2017	Heart failure, sinus rhythm, and coronary disease	Thienopyridine (35%)	Rivaroxaban 2.5 mg twice daily + aspirin 100 mg once daily	2,507	66.5 ± 10.1	78%	Median 21.1 months	Composite of death from any cause, MI, or stroke
						Placebo + aspirin 100 mg once daily	2,515	66.3 ± 10.3	76%		
COMMANDER HF^b^ Greenberg et al. (2019)	2019	32 (628)	Sep 2013 to Oct 2017	Heart failure, sinus rhythm, and coronary disease	Thienopyridine (35%)	Rivaroxaban 2.5 mg twice daily + aspirin 100 mg once daily	2,507	66.5 ± 10.1	78%	Median 21.1 months	Thromboembolic events
						Placebo + aspirin 100 mg once daily	2,515	66.3 ± 10.3	76%		
ATLAS ACS 2-TIMI 51 Mega et al. (2012)	2012	44 (766)	Nov 2008 to Sep 2011	ACS within 7 days	Thienopyridine (clopidogrel or ticlopidine) (93%)	Rivaroxaban 2.5 mg twice daily + aspirin 75–100 mg once daily	5,174	61.8 ± 9.2	75%	Mean 13.1 months	Composite of CV, MI, or stroke (ischemic, hemorrhagic, or stroke of uncertain cause)
						Rivaroxaban 5 mg twice daily+ aspirin 75–100 mg once daily	5,176	61.9 ± 9.0	74%		
						Placebo+ aspirin 75–100 mg once daily	5,176	61.5 ± 9.4	75%		
ATLAS ACS-TIMI 46 Mega et al. (2009)	2009	27 (297)	Nov 2006 to Sep 2008	ACS within 7 days	Thienopyridine (76%)	Rivaroxaban 5 mg daily + aspirin 75–100 mg daily	307	57.2 ± 9.5	78%	6 months	Composite of death, MI, stroke, or severe recurrent ischemia requiring revascularization
						Rivaroxaban 10 mg daily + aspirin 75-100 mg daily	1,056				
						Placebo + aspirin 75–100 mg daily	1,160	57.8 ± 9.6	76%		
GEMINI-ACS-1 Ohman et al. (2017)	2017	21 (371)	Apr 2015 to Oct 2016	ACS (<10 days)	Clopidogrel or ticagrelor (100%)	Rivaroxaban 2.5 mg twice daily	1,519	62.0 (57.0 to 69.0)	75%	Median 291 days	Composite of CV, MI, stroke, or definite stent thrombosis
						Aspirin 100 mg once daily	1,518	63.0 (57.0 to 69.0)	75%		

^a^ Clopidogrel could be administered for up to 6 months after revascularization at the discretion of the investigator;

^b^ A Post Hoc analysis of the COMMANDER HF trial;

^c^ Presented as Median (IQR) or mean ± standard deviation.

CV, cardiovascular death; MI, myocardial infarction; ACS, acute coronary syndrome; CAD, coronary artery disease; PAD, peripheral artery disease; VOYOGER PAD, Vascular Outcomes Study of ASA (acetylsalicylic acid) Along with Rivaroxaban in Endovascular or Surgical Limb Revascularization for peripheral artery disease; COMPASS, Cardiovascular Outcomes for People Using Anticoagulation Strategies; COMMANDER HF, A Study to Assess the Effectiveness and Safety of Rivaroxaban in Reducing the Risk of Death, Myocardial Infarction, or Stroke in Participants with Heart Failure and Coronary Artery Disease Following an Episode of Decompensated Heart Failure; ATLAS ACS2-TIMI 51, Anti-Xa Therapy to Lower Cardiovascular Events in Addition to Standard Therapy in Subjects with Acute Coronary Syndrome-Thrombolysis in Myocardial Infarction 5; ATLAS ACS- TIMI 46, Rivaroxaban in Combination With Aspirin Alone or With Aspirin and a Thienopyridine in Patients With Acute Coronary Syndromes; GEMINI ACS-1, Clinically Significant Bleeding with Low-Dose Rivaroxaban versus Aspirin, in Addition to P2Y12 Inhibition, in Acute Coronary Syndromes.

All 9 articles (Mega et al., 2009; Mega et al., 2012; Eikelboom et al., 2017; Ohman et al., 2017; Anand et al., 2018; Connolly et al., 2018; Zannad et al., 2018; Greenberg et al., 2019; Bonaca et al., 2020) described the randomization component in the sequence generation process. The ATLAS ACS 2-TIMI 51 trial (Mega et al., 2012) did not specify whether allocation concealment was completed, and we considered that trial to have an unclear risk. It is to be noted that double-blind methods were used in all studies. For the ATLAS ACS-TIMI 46 trial (Mega et al., 2009), the number of patients withdrawing was unbalanced between rivaroxaban and comparator groups during follow-up, so we considered this trial as high risk in the incomplete outcome data domain. The GEMINI-ACS-1 (Ohman et al., 2017) and VOYAGER PAD trials (Bonaca et al., 2020) did not report MACEs as defined in this present review and only individually listed the CV deaths, MIs, and strokes; thus, we believe these trials are high risk in the field of selective reporting. Although pharmaceutical companies act as trial sponsors, most statistical analyses are conducted independently by researchers.

### Meta-Analysis Results

#### Rivaroxaban Plus Aspirin Versus Aspirin Alone

##### CAD

**FIGURE 2:**
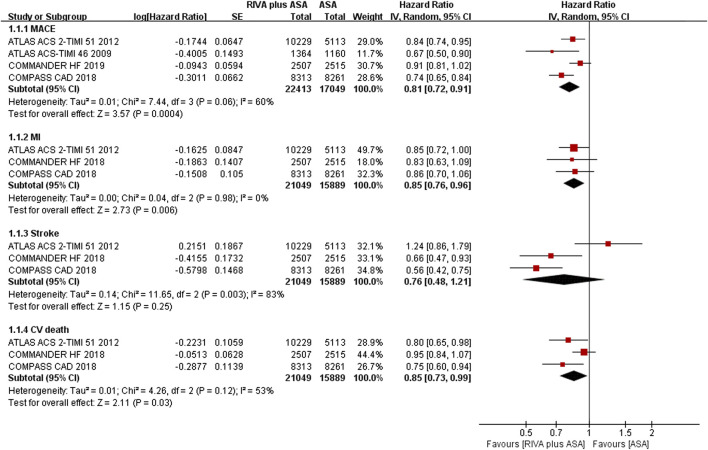
Forest plot of the risk of MACEs in coronary artery disease patients treated with rivaroxaban plus aspirin and aspirin. MACE, major adverse cardiovascular event; MI, myocardial infarction; CV, Cardiovascular; RIVA, rivaroxaban; ASA, aspirin; IV, inverse variance; COMPASS CAD, coronary artery disease in the Cardiovascular Outcomes for People Using Anticoagulation Strategies trial; COMMANDER HF, A Study to Assess the Effectiveness and Safety of Rivaroxaban in Reducing the Risk of Death, Myocardial Infarction, or Stroke in Participants with Heart Failure and Coronary Artery Disease Following an Episode of Decompensated Heart Failure; ATLAS ACS2-TIMI 51, Anti-Xa Therapy to Lower Cardiovascular Events in Addition to Standard Therapy in Subjects with Acute Coronary Syndrome-Thrombolysis in Myocardial Infarction 5; ATLAS ACS- TIMI 46, Rivaroxaban in Combination With Aspirin Alone or With Aspirin and a Thienopyridine in Patients With Acute Coronary Syndromes.

MACEs were reported in 4 articles (Mega et al., 2009; Mega et al., 2012; Connolly et al., 2018; Zannad et al., 2018), of which 22,534 patients reported 1,551 MACEs in the combination group, and 17,112 patients reported 1,482 MACEs in the aspirin group. The meta-analysis indicated that rivaroxaban plus aspirin could reduce risk of MACEs compared with aspirin alone (6.9% vs. 8.7%, HR 0.81, 95% CI, 0.72 to 0.91, *p* = 0.0004, I^2^ = 60%) (Figure 2). Regarding the three MACE components, adding rivaroxaban to aspirin resulted in a lower incidence of MI (3.1% vs. 3.4%, HR 0.85, 95% CI, 0.76 to 0.96, *p* = 0.006, I^2^ = 0%) and CV death (3.9% vs. 5.0%, HR 0.85, 95% CI, 0.73 to 0.99, *p* = 0.03, I^2^ = 53%) than aspirin alone (Mega et al., 2012; Connolly et al., 2018; Zannad et al., 2018). There was no remarkable decrease in risk of stroke (1.1% vs. 1.5%, HR 0.76, 95% CI, 0.48 to 1.21, *p* = 0.25, I^2^ = 83%) (Mega et al., 2012; Connolly et al., 2018; Zannad et al., 2018) (Figure 2).

**FIGURE 3:**
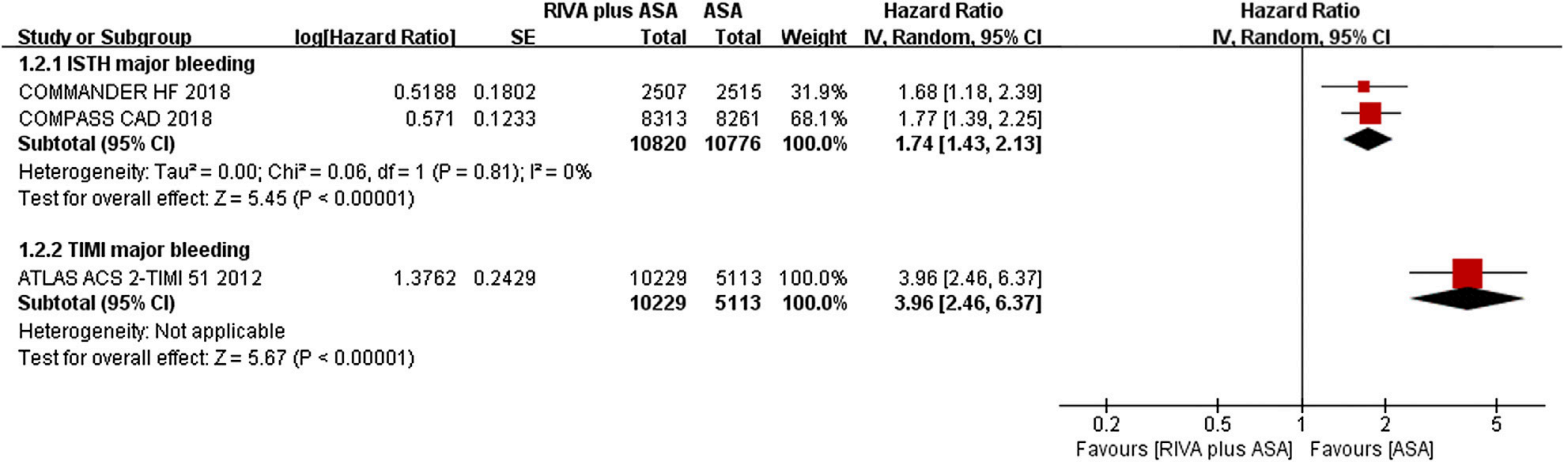
Forest plot of the risk of major bleeding in coronary artery disease patients treated with rivaroxaban plus aspirin and aspirin. ISTH, International Society on Thrombosis and Haemostasis; TIMI, Thrombolysis in Myocardial Infarction; RIVA, rivaroxaban; ASA, aspirin; IV, inverse variance; COMPASS CAD, coronary artery disease in the Cardiovascular Outcomes for People Using Anticoagulation Strategies trial; COMMANDER HF, A Study to Assess the Effectiveness and Safety of Rivaroxaban in Reducing the Risk of Death, Myocardial Infarction, or Stroke in Participants with Heart Failure and Coronary Artery Disease Following an Episode of Decompensated Heart Failure; ATLAS ACS2-TIMI 51, Anti-Xa Therapy to Lower Cardiovascular Events in Addition to Standard Therapy in Subjects with Acute Coronary Syndrome-Thrombolysis in Myocardial Infarction 5.

Two studies (Connolly et al., 2018; Zannad et al., 2018) reported ISTH major bleeding in the CAD population, and the meta-analysis results demonstrated that the addition of rivaroxaban to aspirin could significantly increase incidence of major bleeding when compared aspirin alone (2.5% vs. 1.4%, HR 1.74, 95% CI, 1.43 to 2.13, *p* < 0.00001, I^2^ = 0%). An additional study (Mega et al., 2012) indicated that the combination of rivaroxaban also significantly increased the risk of TIMI major bleeding (2.1% vs. 0.6%, HR 3.96, 95% CI, 2.46 to 6.37, *p* < 0.00001) (Figure 3).

##### PAD

**FIGURE 4:**
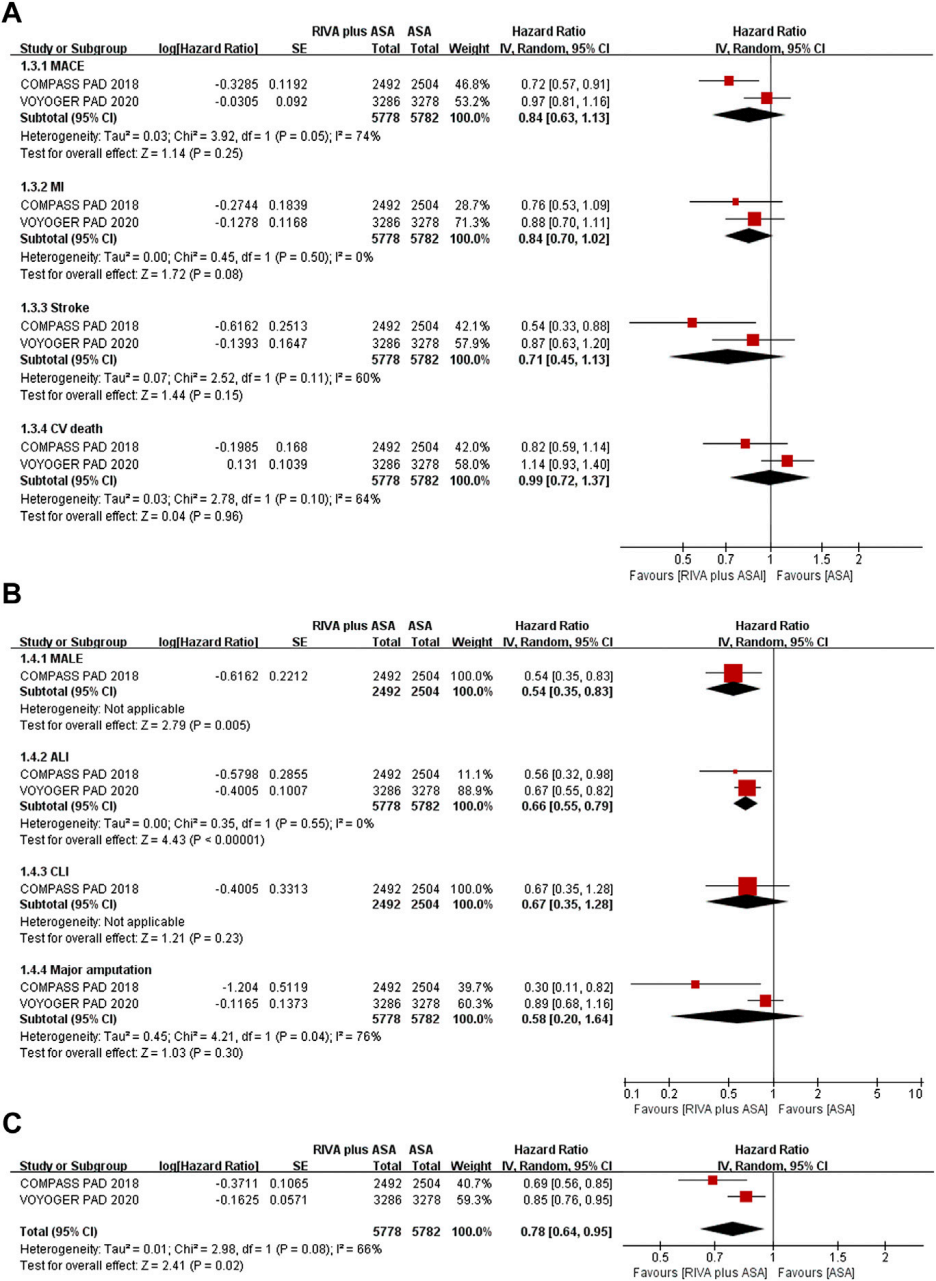
**(A)** Forest plot of the risk of MACE **(B)** Forest plot of the risk of MALE **(C)** Forest plot of the risk of the composite of MACE or MALE in peripheral arterial disease patients treated with rivaroxaban plus aspirin and aspirin. MACE, major adverse cardiovascular event; MI, myocardial infarction; CV, Cardiovascular; MALE, major adverse limb event; ALI, acute limb ischemia; CLI, chronic limb ischemia; RIVA, rivaroxaban; ASA, aspirin; IV, inverse variance; VOYOGER PAD, Vascular Outcomes Study of ASA (acetylsalicylic acid) Along with Rivaroxaban in Endovascular or Surgical Limb Revascularization for peripheral artery disease; COMPASS PAD, peripheral arterial disease in the Cardiovascular Outcomes for People Using Anticoagulation Strategies trial.

Two trials studied PAD patients (Anand et al., 2018; Bonaca et al., 2020), and reductions in MACEs occurrence (9.1% vs. 10.0%, HR 0.84, 95% CI, 0.63 to 1.13, *p* = 0.25, I^2^ = 74%), MI (3.1% vs. 3.7%, HR 0.84, 95% CI, 0.70 to 1.02, *p* = 0.08, I^2^ = 0%), stroke (1.7% vs. 2.2%, HR 0.71, 95% CI, 0.45 to 1.13, *p* = 0.15, I^2^ = 60%), and CV death (4.6% vs. 4.4%, HR 0.99, 95% CI, 0.72 to 1.37, *p* = 0.96, I^2^ = 64%) in the rivaroxaban plus aspirin group were not significant compared with those in the aspirin group (Figure 4A).

Rivaroxaban combined with aspirin significantly lowered incidence of MALEs (0.6% vs. 2.4%, HR 0.54, 95% CI, 0.35 to 0.83, *p* = 0.005) (Anand et al., 2018) and ALI (3.0% vs. 4.5%, HR 0.66, 95% CI, 0.55 to 0.79, *p* < 0.00001, I^2^ = 0%) (Anand et al., 2018; Bonaca et al., 2020) compared with aspirin alone (Figure 4B). Moreover, CLI risk (0.6% vs. 1.0%, HR 0.67, 95% CI, 0.35 to 1.28, *p* = 0.23) (Anand et al., 2018) and major amputation (1.9% vs. 2.1%, HR 0.58, 95% CI, 0.20 to 1.64, *p* = 0.30, I^2^ = 76%) (Anand et al., 2018; Bonaca et al., 2020) were comparable between the combination and aspirin groups (Figure 4B).

Patients receiving rivaroxaban plus aspirin experienced lower risk of composites of MACEs or MALEs than those receiving aspirin alone (11.5% vs. 14.0%, HR 0.78, 95% CI, 0.64 to 0.95, *p* = 0.02, I^2^ = 66%) (Figure 4C) (Anand et al., 2018; Bonaca et al., 2020).

**FIGURE 5:**
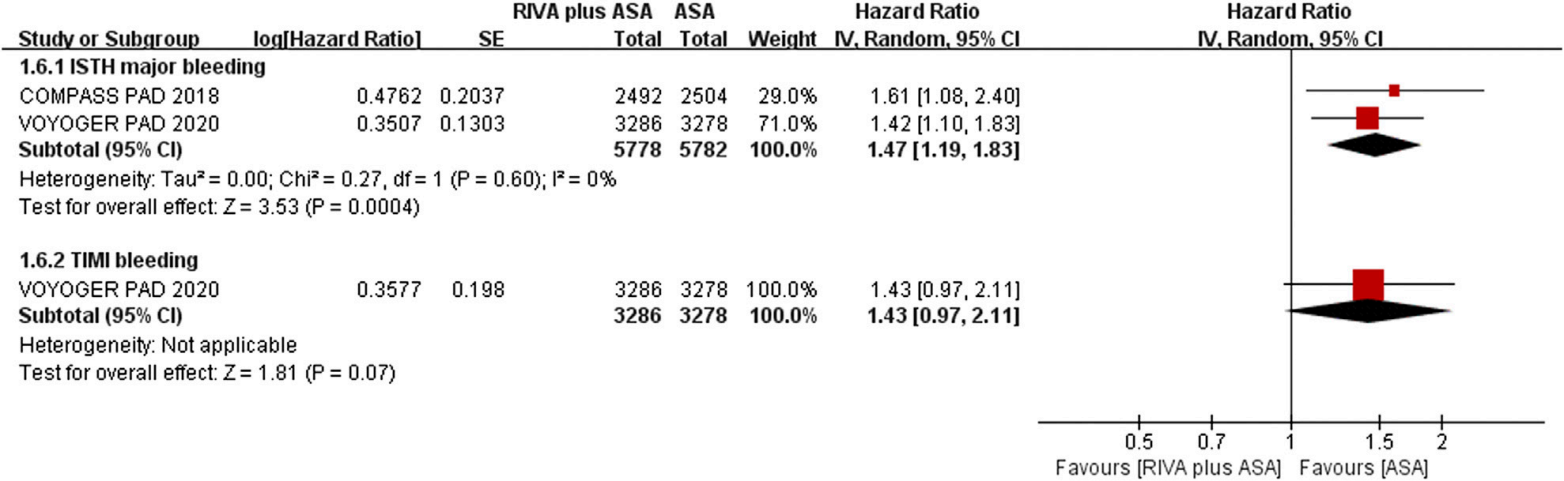
Forest plot of the risk of major bleeding in peripheral arterial disease patients treated with rivaroxaban plus aspirin and aspirin. ISTH, International Society on Thrombosis and Haemostasis; TIMI, Thrombolysis in Myocardial Infarction; RIVA, rivaroxaban; ASA, aspirin; IV, inverse variance VOYOGER PAD, Vascular Outcomes Study of ASA (acetylsalicylic acid) Along with Rivaroxaban in Endovascular or Surgical Limb Revascularization for peripheral artery disease; COMPASS PAD, peripheral arterial disease in the Cardiovascular Outcomes for People Using Anticoagulation Strategies trial.

Two articles (Anand et al., 2018; Bonaca et al., 2020) reported ISTH major bleeding when comparing rivaroxaban plus aspirin versus aspirin alone, and event rate of major bleeding was 2.2% (126/5,778) and 1.5% (84/5,782), separately. The meta-analysis results suggested that the rivaroxaban combination group had a substantially higher incidence of ISTH major bleeding than the aspirin alone group (HR 1.47, 95% CI, 1.19 to 1.83, *p* = 0.0004, I^2^ = 0%) (Figure 5). Rivaroxaban with aspirin (4.3% vs. 3.1%, HR 1.43, 95% CI, 0.97 to 2.11, *p* = 0.07, 1 studies) (Anand et al., 2018) increased risk of TIMI major bleeding over aspirin alone; however, the result was not statistically significant (Figure 5).

#### Rivaroxaban Alone Versus Aspirin Alone

##### CAD

Two articles (Ohman et al., 2017; Connolly et al., 2018) representing 19,548 patients reported benefits of rivaroxaban over aspirin with or without P2Y12 inhibitors, with no significant reduction in risks of MACE, MI, stroke and CV death in CAD patients receiving rivaroxaban versus those receiving aspirin (Supplementary Table S4). This was accompanied by a significant increase in ISTH major bleeding and no significant increase in TIMI major bleeding (Supplementary Table S4).

##### PAD

Only one article (Anand et al., 2018) which enrolled 4,978 PAD patients reported comparisons between rivaroxaban alone versus aspirin alone, and among the two groups, there was no significant difference in incidences of MACE, MI, stroke, CV death, MALE, ALI, CLI, major amputation and the composite of MACEs or MALEs (Supplementary Table S4). Additionally, risk of ISTH major bleeding was also similar between both groups (Supplementary Table S4).

### Sensitivity Analysis

Of all pre-defined primary outcomes, only MACEs (from the CAD group when comparing rivaroxaban plus aspirin to aspirin alone) was reported by three or more studies. We grouped CAD population studies that reported MACEs by diagnosis, rivaroxaban dosage and antiplatelet regimen, and based on the different diagnosis, results were consistent with the primary analysis excluding stable CAD subgroup analysis results (8.2% vs. 9.7%, HR 0.82, 95% CI, 0.67 to 1.01, *p* = 0.06, I^2^ = 82%, 2 studies) (Connolly et al., 2018; Zannad et al., 2018). Subgroup analyses of the incidence of MACE reported by three or more works can be seen in Table 2.

**TABLE 2:** Subgroup analyses of MACE risk in CAD^a^.

Subgroups	No. of studies	Event rate		Heterogeneity	Effect estimate
		Rivaroxaban plus aspirin	Aspirin		
Divided by diagnosis					
ACS Mega et al. (2009), Mega et al. (2012)	2	677/11,714 (5.8%)	438/6,336 (6.9%)	I^2^ = 0%, *p* = 0.56	HR 0.82, 95% CI, 0.74 to 0.91, *p* = 0.0002
Stable CAD Zannad et al. (2018), Connolly et al. (2018)	2	884/10,820 (8.2%)	1,044/10,776 (9.7%)	I^2^ = 82%, *p* = 0.02	HR 0.82, 95% CI, 0.67 to 1.01, *p* = 0.06
Divided by rivaroxaban dosage					
Rivaroxaban 5 mg daily Mega et al. (2009), Mega et al. (2012), Zannad et al. (2018), Connolly et al. (2018)	4	1,218/16,242 (7.5%)	1,499/17,049 (8.8%)	I^2^ = 47%, *p* = 0.13	HR 0.82, 95% CI, 0.74 to 0.92, *p* = 0.0008
Rivaroxaban 10 mg daily Mega et al. (2009), Mega et al. (2012)	2	362/6,171 (5.9%)	455/6,273 (7.3%)	I^2^ = 43%, *p* = 0.19	HR 0.78, 95% CI, 0.62 to 1.00, *p* = 0.05
Divided by antiplatelet regimen					
Aspirin Mega et al.(2009), Mega et al. (2012), Zannad et al. (2018), Connolly et al. (2018)	4	819/10,782 (7.6%)	940/10,374 (9.1%)	I^2^ = 75%, *p* = 0.007	HR 0.79, 95% CI, 0.63 to 0.99, *p* = 0.04
Aspirin plus ticlopidine Mega et al. (2009), Mega et al.(2012), Zannad et al. (2018)	3	786/11,453 (6.9%)	581/6,506 (8.9%)	I^2^ = 0%, *p* = 0.70	HR 0.84, 95% CI, 0.75 to 0.93, *p* = 0.001

^a^ In the comparison of rivaroxaban plus aspirin versus aspirin alone.

ACS, acute coronary syndrome; CAD, coronary artery disease; HR, hazard ratio; CI, confidence interval.

The sensitivity analysis was consistent with the main analysis for MACEs reported by three or more studies when we changed the effect models. Furthermore, sensitivity analysis was performed by individually eliminating studies for primary outcomes, and zero variations were found (Supplementary Table S5).

### Quality of the Evidence

We judged quality of evidence as moderate (downgraded by 1) for all primary and key outcomes except for ISTH major bleeding in PAD patients when comparing rivaroxaban plus aspirin versus aspirin alone, and quality of evidence for the exceptional outcome was high. The GRADE Working Group grades of the primary and key outcomes evidence and downrating explanations are presented in Supplementary Tables S6, S7. The NNT refers to the absolute effect of each outcome in the Supplementary Tables S6, S7.

## Discussion

Our study suggests that rivaroxaban plus aspirin could reduce the incidence of MACE, MI, and CV death, while increasing risk of ITSH major bleeding over aspirin alone for CAD suffers. Rivaroxaban with aspirin groups were significantly associated with decreased risk of MALE, ALI, and the composite of MACE or MALE, and an increase risk of ITSH major bleeding in PAD patients regardless P2Y12 inhibitor use. When comparing rivaroxaban to aspirin, there was no significant decrease in predefined CV and limb events for all ASCVD but there was increased ISTH major bleeding in CAD patients. Whether the ASCVD patients were given P2Y12 inhibitors did not affect the results. The quality of evidence was moderate for all primary and key outcomes except for ISTH major bleeding in PAD.

There are currently three major pathways amplifying platelet activation including the COX-1 pathway, ADP-P2Y12 pathway, and thrombin pathway (Gurbel and Tantry, 2010). Thrombin pathway activity can be reduced by inhibition of thrombin generation via targeting Factor Xa, and inhibition of the platelet protease activated receptor (PAR)-1 (the thrombin receptor) (Gurbel and Tantry, 2010). Additionally, previous data (Zhou et al., 2011; Spronk et al., 2014; Bhatt et al., 2020) demonstrated that the noncanonical mechanisms underlying the potential vascular protective effects of low-dose rivaroxaban included modulation of cellular function either directly by inhibiting effects of factor Xa on PAR-2, or indirectly by inhibiting thrombin generation which may activate PAR-1.

To our knowledge, the ATLAS ACS 2-TIMI 51 trial (Mega et al., 2012) was the first to demonstrate that, for ACS patients, rivaroxaban could lower CV events in combination with aspirin with or without P2Y12 inhibitor. In contrast, the APPRAISE-2 trial (Alexander et al., 2011) indicated that apixaban 5 mg twice a day combined with antiplatelet agents in ACS subjects increased incidence of intracranial hemorrhage and major bleeding without reducing ischemic events, leading to premature termination of the trial. In the subgroup analysis of an earlier systematic review (Oldgren et al., 2013), for ACS patients, adding apixaban (5 mg twice a day) or rivaroxaban (2.5 mg or 5 mg twice a day) to antiplatelet agents was associated with a reduction in incidence of MACE by an average of 15%. This review also showed that rivaroxaban 2.5 or 5 mg twice daily combined with antiplatelet agents could reduce risk of MACE by 19% at an expense of 74% increased risk of ISTH major bleeding compared with aspirin alone in CAD patients.

For PAD suffers, this review revealed that risk of MACE was comparable between the rivaroxaban plus aspirin group and aspirin alone group, but it must be noted should note that there were only two trials (Anand et al., 2018; Bonaca et al., 2020) reported MACE, and the findings of both were contradictory. The COMPASS PAD trial, which enrolled a wide range of PAD participants including symptomatic or asymptomatic lower extremity PAD and carotid artery diseases, found that low dosage rivaroxaban together with aspirin had advantages over aspirin alone for the reduction of MACE risk in overall PAD (Anand et al., 2018). The VOYAGER PAD trial (Bonaca et al., 2020), which only enrolled patients undergoing successful revascularization operations performed in the previous 10 days, did not support the findings that low-dose rivaroxaban combined with aspirin had a lower incidence of MACE than aspirin. This outcome of the VOYAGER PAD trial (Bonaca et al., 2020) agrees to the results of the COMPASS PAD trial (Anand et al., 2018) for the lower extremity PAD subgroup. In addition to MACE, both trials (Anand et al., 2018; Bonaca et al., 2020) focused on limb events outcomes which are critical for PAD patients. The COMPASS PAD trial (Anand et al., 2018) suggested that the addition of low-dose rivaroxaban to aspirin could significantly reduce MALE by approximately 46%, and particularly for improvement of the ALI. Meta-analysis results of the two trials (Anand et al., 2018; Bonaca et al., 2020) indicated that the addition of rivaroxaban clearly decreased the risk of the composite of MACE or MALE by 22% (Anand et al., 2018; Bonaca et al., 2020). Moreover, the COMPASS PAD trial’s (Anand et al., 2018) analysis of the lower extremity PAD subgroups supported the VOYAGER PAD trial (Bonaca et al., 2020), reporting that the effects of low-dose rivaroxaban plus aspirin on the composite of MACE or MALE were significantly better than aspirin alone.

In comparison of rivaroxaban plus aspirin versus aspirin, the risk of ISTH major bleeding increased by 74% in CAD and 47% in PAD; the incidence of TIMI major bleeding was approximately tripled in CAD, while the increase was not significant in PAD. Findings from post hoc analysis of the COMPASS trial (Eikelboom et al., 2019) suggested that the gastrointestinal (GI) tract was the most common site of major bleeding in patients randomized to the combination of rivaroxaban and aspirin. Although several studies (Eikelboom et al., 2017; Zannad et al., 2018; Bonaca et al., 2020) pointed out that the combination of rivaroxaban and aspirin compared with aspirin did not significantly increase fatal bleeding, it may increase major bleeding leading to hospitalization or admittance to an acute care facility without overnight stay (Eikelboom et al., 2019).

Findings of the subgroup analysis suggested the risk of MACE in stable CAD patients was inconsistent when comparing rivaroxaban plus aspirin to aspirin alone with the main analysis. This subgroup included two trials (Connolly et al., 2018; Zannad et al., 2018): the COMPASS-CAD trial (Connolly et al., 2018) found that the addition of rivaroxaban could significantly reduce MACE, while the COMMANDER-HF trial (Zannad et al., 2018) did not obtain a positive result. A post hoc analysis (Greenberg et al., 2019) of the COMMANDER HF trial suggested the possibility that the difference in incidence of MACEs between the COMMANDER HF trial (Zannad et al., 2018) and COMPASS CAD trial (Connolly et al., 2018) was related to subjects exhibiting recently worsened chronic heart failure in the former trial, and the higher rate of CV deaths in COMMANDER HF trial (Zannad et al., 2018) were related to pump failure that were not responsible for antithrombotic treatment, masking a favorable association between rivaroxaban and MACEs. Furthermore, given the high statistical heterogeneity (Higgins et al., 2003), the results of the two studies should be treated with caution in clinical practice. MACE risk both reduced in two doses of the rivaroxaban group, but the rates of TIMI major bleeding were lower in patients receiving 5-mg doses than those receiving 10-mg dose and a survival benefit that was not seen with the 10-mg dose (Mega et al., 2013; Eikelboom et al., 2017). When eligible CAD patients was grouped by antiplatelet regimen, the findings showed that regardless of adding rivaroxaban to single antiplatelet (SAPT) or dual antiplatelet (DAPT), MACE risk reduction both were significant compared with standard SAPT or DAPT. Nevertheless, risk of ISTH major bleeding in rivaroxaban together with DAPT (at a course of approximately 13 months) was three to four times higher than standard DAPT. About half of the patients enrolled in the VOYAGER PAD trial (Bonaca et al., 2020) were administrated a short course (≤6 months) of rivaroxaban concomitant DAPT, which should be noted that the use of DAPT was not randomized in comparison. A newly published article based on VOYAGER PAD trial (Hiatt et al., 2020) has suggested that rivaroxaban plus aspirin reduces the risk of the composite of MACE or MALE regardless of clopidogrel use, and the safety of rivaroxaban was consistent regardless of clopidogrel use, however a trend of increased ISTH major bleeding with clopidogrel use was seen >30 days over a shorter duration.

This review suggests that all the cardiovascular or limb ischemic events were not significantly reduced, and major bleeding may increase, when comparing rivaroxaban alone versus aspirin alone with or without P2Y12 inhibitors. One (Ohman et al., 2017) of two trials (Eikelboom et al., 2017; Ohman et al., 2017) included in this comparison investigated a new strategy of using rivaroxaban instead of clopidogrel in addition to aspirin. It is anticipated that this strategy will balance the risks of ischemia and bleeding and achieve maximum clinical net benefit. However, current small sample size studies in ACS (Ohman et al., 2017) and PAD (Moll et al., 2018; Jetty et al., 2019) are not sufficient to assess the effect on ischemic events.

The present review gave a comprehensive analysis of the role of low-dose rivaroxaban in ASCVD including ACS, stable CAD, and PAD, and the findings supported that rivaroxaban 5 mg daily dose plus aspirin may be beneficial for ASCVD patients regardless of added concomitant ticlopidine. However, for patients with higher risk of GI bleeding, it should be used after weighing the risks and benefits in clinical practice. Moreover, if practitioners choose to use the triple antithrombotic regimen (low-dose rivaroxaban plus DAPT), the current evidence inspired that the course should shorten as much as possible, and a short course of ≤30 days is recommended for lower extremity PAD.

There were several unavoidable limitations in this review. First, all studies were funded by pharmaceutical companies, but most of the data analyses were done independently by the researchers. Second, with the limited number of studies, subgroup analysis of the included studies could not be carried out for most outcomes according to daily doses of rivaroxaban, antiplatelet regimen, and follow-up time, which may be more suited for the individualized patient treatments. Third, the PAD patients included in this study did not include the patients with carotid artery disease after recent revascularization; therefore, it was not able to provide direct evidence for the use of low-dose rivaroxaban after recent carotid artery disease revascularization. Fourth, the primary outcome was a composite of multiple systemic and limb-related outcomes, which is challenging to connect with clinical practice. Fifth, the most beneficial duration of the combination of rivaroxaban and standard antiplatelet regimens has not yet been determined.

## Conclusion

Compared to standard antiplatelet therapy, the addition of rivaroxaban 5 mg daily dose to standard antiplatelet therapy may improve the cardiovascular or limb outcomes of ASCVD patients, with an increase in major bleeding. Patients who would benefit from the addition of low-dose rivaroxaban to antiplatelet agents and the appropriate course of dual-pathway antithrombotic strategies should be identified in clinical practice to individualize antithrombotic therapy. For future study, more reports including RCTs and real-world studies are still needed to verify the efficacy and safety of dual-pathway antithrombotic strategies for ASCVD.

## Data Availability

The original contributions presented in the study are included in the article/Supplementary Material, further inquiries can be directed to the corresponding authors.
