# Supplementary material for: Low Dose Rivaroxaban for Atherosclerotic Cardiovascular Diseases: A Systematic Review and Meta-analysis
Source: Front Pharmacol. 2021 Feb 8;11:608247. doi: 10.3389/fphar.2020.608247 (PMC7957832; doi:10.3389/fphar.2020.608247)
Supplement: Supplementary file 1 [file table1.docx]

Supplementary materials

Table S1 Queries in PubMed

Table S2 Queries in Cochrane Library

Table S3 Queries in Embase

Table S4 Meta-analysis of the incidence of the predefined outcomes in rivaroxaban alone and aspirin alone groups

Table S5 The sensitivity analysis results of eliminating each study individually for primary outcomes

Table S6 GRADE Working Group grades of evidence for primary outcomes in the comparison of rivaroxaban plus aspirin versus aspirin alone

Table S7 GRADE Working Group grades of evidence for primary outcomes in the comparison of rivaroxaban alone versus aspirin alone

Figure S1 Risk of bias graph

“Green” represents low risk of bias, “Red” represents high risk of bias, and “Yellow” represents unclear risk of bias.

Figure S2 Risk of bias summary

“Green” represents low risk of bias, “Red” represents high risk of bias, and “Yellow” represents unclear risk of bias. VOYOGER PAD: Vascular Outcomes Study of ASA (acetylsalicylic acid) Along with Rivaroxaban in Endovascular or Surgical Limb Revascularization for peripheral artery disease; COMPASS: Cardiovascular Outcomes for People Using Anticoagulation Strategies; COMPASS-PAD: peripheral artery disease in the COMPASS trial; COMPASS-CAD: coronary artery disease in the COMPASS trial; COMMANDER HF: A Study to Assess the Effectiveness and Safety of Rivaroxaban in Reducing the Risk of Death, Myocardial Infarction, or Stroke in Participants with Heart Failure and Coronary Artery Disease Following an Episode of Decompensated Heart Failure; ATLAS ACS2-TIMI 51: Anti-Xa Therapy to Lower Cardiovascular Events in Addition to Standard Therapy in Subjects with Acute Coronary Syndrome-Thrombolysis in Myocardial Infarction 5; ATLAS ACS- TIMI 46: Rivaroxaban in Combination With Aspirin Alone or With Aspirin and a Thienopyridine in Patients With Acute Coronary Syndromes; GEMINI ACS-1: Clinically Significant Bleeding with Low-Dose Rivaroxaban versus Aspirin, in Addition to P2Y12 Inhibition, in Acute Coronary Syndromes.

Table S1 Queries in PubMed

| Search | Query | Items found |
| --- | --- | --- |
| #50 | Search ((((((((((((("Rivaroxaban"[Mesh]) OR "apixaban" [Supplementary Concept]) OR "edoxaban" [Supplementary Concept])) OR "Factor Xa Inhibitors"[Mesh]) OR Xarelto[Title/Abstract]) OR Eliquis[Title/Abstract]) OR ((((((Factor Xa Inhibitors[Title/Abstract]) OR Direct Factor Xa Inhibitors[Title/Abstract]) OR Direct-Acting Oral Anticoagulants[Title/Abstract]) OR Anticoagulants, Direct-Acting Oral[Title/Abstract]) OR Direct Acting Oral Anticoagulants[Title/Abstract]) OR Oral Anticoagulants, Direct-Acting[Title/Abstract]))) OR Xarelto[Title/Abstract]) OR Eliquis[Title/Abstract]) OR (((Rivaroxaban[Title/Abstract]) OR apixaban[Title/Abstract]) OR edoxaban[Title/Abstract]))) AND ((((((((("Peripheral Arterial Disease"[Mesh] OR "Arterial Occlusive Diseases"[Mesh] OR "Carotid Artery Diseases"[Mesh] OR "Peripheral Arterial Occlusive Disease 1" [Supplementary Concept]) OR "Coronary Artery Disease"[Mesh])) OR "Coronary Disease"[Mesh]) OR "Carotid Stenosis"[Mesh])) OR "Acute Coronary Syndrome"[Mesh])) OR (((((((((((Peripheral Arterial Disease[Title/Abstract]) OR Peripheral Artery Disease?[Title/Abstract]) OR Carotid Artery Disease?[Title/Abstract]) OR Carotid Arterial Disease?[Title/Abstract]) OR Carotid Atherosclerosis[Title/Abstract]) OR Carotid Atheroscleroses[Title/Abstract]) OR Carotid Artery Disorder?[Title/Abstract]) OR Carotid Stenosis[Title/Abstract])) OR ((((Coronary Disease?[Title/Abstract]) OR Coronary Heart Disease?[Title/Abstract]) OR Coronary Artery Disease?[Title/Abstract]) OR Coronary Arterial Disease?[Title/Abstract])) OR Acute Coronary Syndrome?[Title/Abstract])) | 743 |

**Table S2 Queries in Cochrane Library**

#1 MeSH descriptor: [Factor Xa Inhibitors] explode all trees

#2 MeSH descriptor: [Rivaroxaban] explode all trees

#3 Rivaroxaban:ti,ab,kw

#4 Xarelto:ti,ab,kw

#5 apixaban:ti,ab,kw

#6 Eliquis:ti,ab,kw

#7 edoxaban:ti,ab,kw

#8 (Factor Xa Inhibitors OR Direct Factor Xa Inhibitors OR Direct-Acting Oral Anticoagulants):ti,ab,kw

#9 {OR #1-#8}

#10 MeSH descriptor: [Peripheral Arterial Disease] explode all trees

#11 MeSH descriptor: [Arterial Occlusive Diseases] explode all trees

#12 MeSH descriptor: [Carotid Artery Diseases] explode all trees

#13 MeSH descriptor: [Carotid Stenosis] explode all trees

#14 MeSH descriptor: [Coronary Disease] explode all trees

#15 MeSH descriptor: [Coronary Artery Disease] explode all trees

#16 MeSH descriptor: [Acute Coronary Syndrome] explode all trees

#17 (Peripheral Arterial Disease? OR Peripheral Artery Disease? OR Carotid Artery Disease? OR Carotid Arterial Disease? OR Carotid Atherosclerosis OR Carotid Atheroscleroses OR Carotid Artery Disorder? OR Carotid Stenosis OR Coronary Disease? OR Coronary Heart Disease? OR Coronary Artery Disease? OR Coronary Arterial Disease? OR Acute Coronary Syndrome?):ti,ab,kw

#18 {OR #10-#17}

#19 #9 AND #18 in Trials

**Table S3 Queries in Embase**

| No. | Query | Results |
| --- | --- | --- |
| #24 | #11 AND #22 AND [humans]/lim AND [english]/lim | 2634 |
| #23 | #11 AND #22 | 2851 |
| #22 | #12 OR #13 OR #14 OR #15 OR #16 OR #17 OR #18 OR #19 OR #20 OR #21 | 569131 |
| #21 | 'carotid arter* disorder*':ab,kw,ti | 11 |
| #20 | 'carotid arter* disease*':ab,kw,ti | 4401 |
| #19 | 'acute coronary syndrome*':ab,kw,ti | 56773 |
| #18 | 'coronary arter* disease*':ab,kw,ti | 141020 |
| #17 | 'peripheral arter* disease*':ab,kw,ti | 23305 |
| #16 | 'carotid arter* occlusion*':ab,kw,ti | 4826 |
| #15 | 'carotid stenosis*':ab,kw,ti | 10298 |
| #14 | 'coronary artery disease'/exp | 336752 |
| #13 | 'carotid artery disease'/exp | 69443 |
| #12 | 'peripheral occlusive artery disease'/exp | 173689 |
| #11 | #1 OR #2 OR #3 OR #4 OR #5 OR #6 OR #7 OR #8 OR #9 OR #10 | 22262 |
| #10 | 'direct-acting oral anticoagulants':ab,kw,ti | 355 |
| #9 | rivaroxaban:ab,kw,ti | 10537 |
| #8 | 'factor xa inhibitors':ab,kw,ti | 1921 |
| #7 | xarelto:ab,kw,ti | 307 |
| #6 | eliquis:ab,kw,ti | 147 |
| #5 | apixaban:ab,kw,ti | 6864 |
| #4 | edoxaban:ab,kw,ti | 2367 |
| #3 | 'edoxaban'/exp | 4386 |
| #2 | 'apixaban'/exp | 11617 |
| #1 | 'rivaroxaban'/exp | 17081 |

**Table S4 Meta-analysis of the incidence of the predefined outcomes in rivaroxaban alone and aspirin alone groups**

| **Population** | **Outcomes** | **No. of studies** | **Event rate** | | **Heterogeneity** | **Effect Estimate** |
| --- | --- | --- | --- | --- | --- | --- |
|  |  |  | **Rivaroxaban** | **aspirin** |  |  |
| CAD (Ohman et al., 2017; Connolly et al.,2018) | MACE | 2 | 487/9769, 5.0% | 532/9779, 5.4% | P=0.32, I2=0% | HR 0.91, 95%CI [0.81, 1.03], P=0.14 |
|  | MI | 2 | 232/9769, 2.4% | 244/9779, 2.5% | P=0.27, I2=18% | HR 0.96, 95%CI [0.78, 1.18], P=0.69 |
|  | Stroke | 2 | 112/9769, 1.1% | 142/9779, 1.5% | P=0.50, I2=0% | HR 0.79, 95%CI [0.61, 1.02], P=0.07 |
|  | CV death | 2 | 194/9769, 2.0% | 201/9779, 2.1% | P=0.64, I2=0% | HR 0.96, 95%CI [0.79, 1.18], P=0.72 |
|  | ISTH major bleeding | 2 | 195/9769, 2.0% | 122/9779, 1.2% | P=0.64, I2=0% | HR 1.61, 95%CI [1.28, 2.01], P<0.0001 |
|  | TIMI major bleeding | 1 | 10/1519, 0.7% | 8/1518, 0.5% | NA | HR 1.25, 95%CI [0.49 3.19], P=0.64 |
| PAD (Anand et al., 2018) | MACE | 1 | 149/2474, 6.0% | 174/2504, 6.9% | NA | HR 0.86, 95%CI [0.69 1.07], P=0.18 |
|  | MI | 1 | 56/2474, 2.3% | 67/2504, 2.7% | NA | HR 0.84, 95%CI [0.59 1.20], P=0.33 |
|  | Stroke | 1 | 43/2474, 1.7% | 47/2504，1.9% | NA | HR 0.93, 95%CI [0.61 1.42], P=0.74 |
|  | CV death | 1 | 66/2474, 2.7% | 78/2504, 3.1% | NA | HR 0.86, 95%CI [0.62 1.19], P=0.37 |
|  | MALE | 1 | 40/2474, 1.6% | 60/2504, 2.4% | NA | HR 0.67, 95%CI [0.45 1.00], P=0.05 |
|  | ALI | 1 | 19/2474, 0.8% | 34/2504, 1.4% | NA | HR 0.57, 95%CI [0.32 1.02], P=0.06 |
|  | CLI | 1 | 18/2474, 0.7% | 24/2504, 1.0% | NA | HR 0.76, 95%CI [0.41 1.41], P=0.38 |
|  | Major amputation | 1 | 8/2474, 0.3% | 17/2504, 0.7% | NA | HR 0.46, 95%CI [0.20 1.06], P=0.07 |
|  | Composite of MACE or MALE | 1 | 188/2474, 7.6% | 225/2504, 9.0% | NA | HR 0.84,95%CI [0.69, 1.02], P=0.08 |
|  | ISTH major bleeding | 1 | 53/2474, 2.1% | 40/2504, 1.6% | NA | HR 1.34,95%CI [0.89, 2.02], P=0.16 |

CAD, coronary artery disease, PAD, peripheral artery disease; MACE, major adverse cardiovascular event; MI, myocardial infarction; CV, cardiovascular; MALE, major limb adverse event; ALI, acute limb ischemia; CLI, chronic limb ischemia; ISTH, International Society on Thrombosis and Haemostasis; TIMI, Thrombolysis in Myocardial Infarction; HR, hazard ratio; CI, confidence interval; NA, not applicable.

**Table S5 The sensitivity analysis results of eliminating each study individually for primary outcomes ^a^**

| **Population** | **Outcome** | **Study removed** | **Heterogeneity** | **Effect estimated** |
| --- | --- | --- | --- | --- |
| CAD | MACE | ATLAS ACS 2-TIMI 51 2012 | P=0.03, I2=73% | HR 0.79, 95% CI (0.66 to 0.94), P=0.008 |
|  |  | ATLAS ACS-TIMI 46 2009 | P=0.07, I2=63% | HR 0.83, 95% CI (0.74 to 0.93), P=0.002 |
|  |  | COMMANDER HF 2018 | P=0.23, I2=33% | HR 0.77, 95% CI (0.69 to 0.86), P<0.00001 |
|  |  | COMPASS CAD 2018 | P=0.15, I2=48% | HR 0.84, 95% CI (0.74 to 0.95), P=0.007 |

^a^ Focus on outcomes reported by three more studies in the comparison of rivaroxaban plus aspirin versus aspirin alone

CAD, coronary artery disease including acute coronary syndrome and stable coronary artery disease; MACE, adverse cardiovascular event; COMPASS CAD, coronary artery disease in Cardiovascular Outcomes for People Using Anticoagulation Strategies trial; COMMANDER HF, A Study to Assess the Effectiveness and Safety of Rivaroxaban in Reducing the Risk of Death, Myocardial Infarction, or Stroke in Participants with Heart Failure and Coronary Artery Disease Following an Episode of Decompensated Heart Failure; ATLAS ACS2-TIMI 51, Anti-Xa Therapy to Lower Cardiovascular Events in Addition to Standard Therapy in Subjects with Acute Coronary Syndrome-Thrombolysis in Myocardial Infarction 5; ATLAS ACS- TIMI 46, Rivaroxaban in Combination With Aspirin Alone or With Aspirin and a Thienopyridine in Patients With Acute Coronary Syndromes; HR, hazard ratio; CI, confidence interval; NA, not applicable.

**Table S6 GRADE Working Group grades of evidence for primary outcomes in the comparison of rivaroxaban plus aspirin versus aspirin alone**

Patient or population: patients with atherosclerotic cardiovascular diseases

Intervention: low dose rivaroxaban plus aspirin

Comparison: aspirin alone

| **Certainty assessment** | | | | | | | **№ of patients** | | **Effect** | | **Certainty** |
| --- | --- | --- | --- | --- | --- | --- | --- | --- | --- | --- | --- |
| **№ of studies** | **Study design** | **Risk of bias** | **Inconsistency** | **Indirectness** | **Imprecision** | **Other considerations** | **Rivaroxaban plus aspirin** | **Aspirin** | **Relative (95% CI)** | **Absolute (95% CI)** |  |
| **CAD - MACE** | | | | | | | | | | | |
| 4 | randomized trials | serious ^a^ | not serious | not serious | not serious | none | 22413 participants | 17049 participants | **HR 0.81** (0.72 to 0.91) [CAD - MACE] | **16 fewer per 1,000** (from 24 fewer to 8 fewer) | ⨁⨁⨁◯ Moderate |
|  |  |  |  |  |  |  | - | 8.7% |  | **16 fewer per 1,000** (from 24 fewer to 8 fewer) |  |
| **CAD - ISTH Major bleeding** | | | | | | | | | | | |
| 2 | randomized trials | not serious | not serious | serious ^b^ | not serious | none | 10820 participants | 10776 participants | **HR 1.74** (1.43 to 2.13) [CAD - ISTH major bleeding] | **10 more per 1,000** (from 6 more to 16 more) | ⨁⨁⨁◯ Moderate |
|  |  |  |  |  |  |  | - | 1.4% |  | **10 more per 1,000** (from 6 more to 16 more) |  |
| **PAD - MACE** | | | | | | | | | | | |
| 2 | randomized trials | not serious | serious ^c^ | not serious | not serious | none | 5778 participants | 5782 participants | **HR 0.84** (0.63 to 1.13) [PAD - MACE] | **15 fewer per 1,000** (from 36 fewer to 12 more) | ⨁⨁⨁◯ Moderate |
|  |  |  |  |  |  |  | - | 10.0% |  | **15 fewer per 1,000** (from 36 fewer to 12 more) |  |
| **PAD - MALE** | | | | | | | | | | | |
| 1 | randomized trials | not serious | not serious | not serious | not serious | publication bias strongly suspected ^d^ | 2492 participants | 2504 participants | **HR 0.54** (0.35 to 0.83) [PAD - MALE] | **11 fewer per 1,000** (from 16 fewer to 4 fewer) | ⨁⨁⨁◯ Moderate |
|  |  |  |  |  |  |  | - | 2.4% |  | **11 fewer per 1,000** (from 16 fewer to 4 fewer) |  |
| **PAD - MACE Or MALE** | | | | | | | | | | | |
| 1 | randomized trials | not serious | not serious | not serious | not serious | publication bias strongly suspected ^d^ | 2492 participants | 2504 participants | **HR 0.69** (0.56 to 0.85) [PAD – MACE or MALE] | **41 fewer per 1,000** (from 59 fewer to 20 fewer) | ⨁⨁⨁◯ Moderate |
|  |  |  |  |  |  |  | - | 14.0% |  | **41 fewer per 1,000** (from 59 fewer to 20 fewer) |  |
| 1 | randomized trials | not serious | not serious | not serious | not serious | publication bias strongly suspected ^d^ | 2492 participants | 2504 participants | **HR 1.61** (1.08 to 2.40) [PAD major bleeding - ISTH major bleeding] | **9 more per 1,000** (from 1 more to 21 more) | ⨁⨁⨁◯ Moderate |
|  |  |  |  |  |  |  | - | 1.5% |  | **9 more per 1,000** (from 1 more to 21 more) |  |

CI: Confidence interval; HR: Hazard Ratio; CAD, coronary artery disease, PAD, peripheral artery disease; MACE, major adverse cardiovascular event; MALE, major limb adverse event; ISTH, International Society on Thrombosis and Haemostasis.

Explanations

a. Two studies included in the meta-analysis had high or unclear risk in key domains through the risk of bias assessment.

b. Meta-analysis of the studies did not cover the ACS patients for studies focused on the ACS patients only report TIMI major bleeding as safety outcomes.

c. Meta-analysis of the two studies for the MACE reported inconsistent results.

d. Only one study included in the meta-analysis for the outcome.

**Table S7 GRADE Working Group grades of evidence for primary outcomes in the comparison of rivaroxaban alone versus aspirin alone**

**Patient or population: patients with atherosclerotic cardiovascular diseases**

**Intervention: low dose rivaroxaban alone**

**Comparison: aspirin alone**

| **Certainty assessment** | | | | | | | **№ of patients** | | **Effect** | | **Certainty** |
| --- | --- | --- | --- | --- | --- | --- | --- | --- | --- | --- | --- |
| **№ of studies** | **Study design** | **Risk of bias** | **Inconsistency** | **Indirectness** | **Imprecision** | **Other considerations** | **Rivaroxaban** | **Aspirin** | **Relative (95% CI)** | **Absolute (95% CI)** |  |
| **CAD - MACE** | | | | | | | | | | | |
| 2 | randomized trials | serious ^b^ | not serious | not serious | not serious | none | 9769 participants | 9779 participants | **HR 0.91** (0.81 to 1.03) [CAD - MACE] | **5 fewer per 1,000** (from 10 fewer to 2 more) | ⨁⨁⨁◯ Moderate |
|  |  |  |  |  |  |  | - | 5.4% |  | **5 fewer per 1,000** (from 10 fewer to 2 more) |  |
| **CAD - ISTH Major bleeding** | | | | | | | | | | | |
| 2 | randomized trials | not serious | not serious | not serious | not serious | none | 9769 participants | 9779 participants | **HR 1.61** (1.28 to 2.01) [CAD - ISTH major bleeding] | **7 more per 1,000** (from 3 more to 12 more) | ⨁⨁⨁⨁ High |
|  |  |  |  |  |  |  | - | 1.2% |  | **7 more per 1,000** (from 3 more to 12 more) |  |
| **PAD - MACE** | | | | | | | | | | | |
| 1 | randomized trials | not serious | not serious | not serious | not serious | publication bias strongly suspected ^a^ | 2474 participants | 2504 participants | **HR 0.86** (0.69 to 1.07) [PAD- MACE] | **9 fewer per 1,000** (from 21 fewer to 5 more) | ⨁⨁⨁◯ Moderate |
|  |  |  |  |  |  |  | - | 6.9% |  | **9 fewer per 1,000** (from 21 fewer to 5 more) |  |
| **PAD - MALE** | | | | | | | | | | | |
| 1 | randomized trials | not serious | not serious | not serious | not serious | publication bias strongly suspected ^a^ | 2474 participants | 2504 participants | **HR 0.67** (0.45 to 1.00) [PAD - MALE] | **8 fewer per 1,000** (from 13 fewer to 0 fewer) | ⨁⨁⨁◯ Moderate |
|  |  |  |  |  |  |  | - | 2.4% |  | **8 fewer per 1,000** (from 13 fewer to 0 fewer) |  |
| **PAD – MACE** or **MALE** | | | | | | | | | | | |
| 1 | randomized trials | not serious | not serious | not serious | not serious | publication bias strongly suspected ^a^ | 2474 participants | 2504 participants | **HR 0.84** (0.69 to 1.02) [PAD – MACE or MALE] | **14 fewer per 1,000** (from 27 fewer to 2 more) | ⨁⨁⨁◯ Moderate |
|  |  |  |  |  |  |  | - | 9.0% |  | **14 fewer per 1,000** (from 27 fewer to 2 more) |  |
| **PAD - ISTH major bleeding** | | | | | | | | | | | |
| 1 | randomized trials | not serious | not serious | not serious | not serious | publication bias strongly suspected ^a^ | 2474 participants | 2504 participants | **HR 1.34** (0.89 to 2.02) [PAD - ISTH major bleeding] | **5 more per 1,000** (from 2 fewer to 16 more) | ⨁⨁⨁◯ Moderate |
|  |  |  |  |  |  |  | - | 1.6% |  | **5 more per 1,000** (from 2 fewer to 16 more) |  |

CI: Confidence interval; HR: Hazard Ratio; CAD, coronary artery disease, PAD, peripheral artery disease; MACE, major adverse cardiovascular event; MALE, major limb adverse event; ISTH, International Society on Thrombosis and Haemostasis

Explanations

a. Only one study included in the meta-analysis for the outcome.

b. The GEMINI-ACS-1 trial was a phase 2 trial, and was not powered to assess the effect on ischemic events.


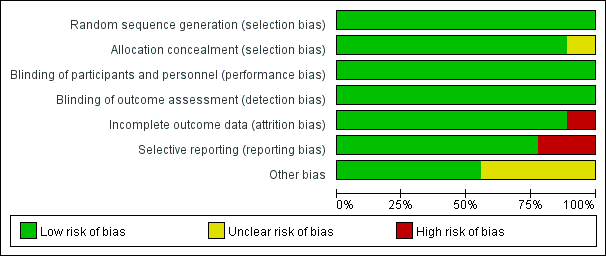


**Figure S1 Risk of bias graph**


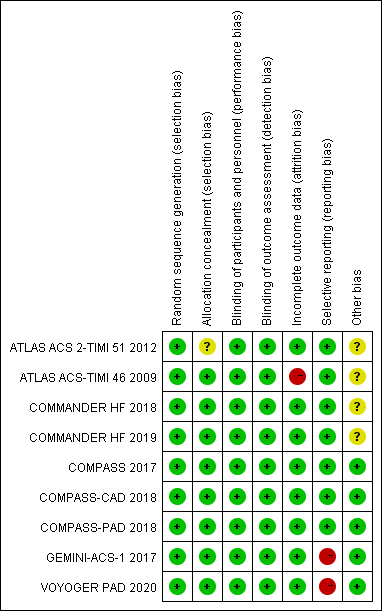


**Figure S2 Risk of bias summary**
